# Suppressive Effect of Black Soldier Fly Larvae Frass on Fusarium Wilt Disease in Tomato Plants

**DOI:** 10.3390/insects15080613

**Published:** 2024-08-15

**Authors:** Ghazaleh Arabzadeh, Maxime Delisle-Houde, Grant W. Vandenberg, Marie-Hélène Deschamps, Martine Dorais, Nicolas Derome, Russell J. Tweddell

**Affiliations:** 1 Département des Sciences Animales, Université Laval, Québec, QC G1V 0A6, Canada; ghazaleh.arabzadeh.1@ulaval.ca (G.A.); grant.vandenberg@fsaa.ulaval.ca (G.W.V.); marie-helene.deschamps@fsaa.ulaval.ca (M.-H.D.); 2 Département de Phytologie, Université Laval, Québec, QC G1V 0A6, Canada; maxime.delisle-houde.1@ulaval.ca (M.D.-H.); martine.dorais@fsaa.ulaval.ca (M.D.); 3 Département de Biologie, Université Laval, Québec, QC G1V 0A6, Canada; nicolas.derome@bio.ulaval.ca

**Keywords:** black soldier fly, frass, Fusarium wilt, tomato, plant disease, soil-borne pathogen

## Abstract

**Simple Summary:**

Black soldier fly larvae (BSFL) frass, the residual material resulting from the bioconversion of organic matter by BSFL, is gaining attention as a potential organic amendment for plant growth. Studies have reported the potential of BSFL frass to replace commercial inorganic fertilizers in food crops. Besides improving soil fertility, BSFL frass was recently shown to have antifungal activity against different fungal plant pathogens, suggesting that it could suppress fungal plant diseases. This study investigated the effect of BSFL frass derived from BSFL reared on a diet composed of fruit/vegetable/bakery/brewery residues (FVBB diet) and on the Gainesville diet (GV diet) on the development of Fusarium wilt in tomato plants caused by the fungus *Fusarium oxysporum* f. sp. *lycopersici* (FOL). The results show that frass from BSFL reared on the GV diet, treated with heat (70 °C, 1 h) or not, inhibited FOL root colonization and reduced the severity of the disease to a far greater extent than frass from BSFL reared on a FVBB diet and commercial compost made of peat, seaweed, and shrimps. This study suggests that BSFL frass, depending on the larval rearing diet, could serve as a soil amendment to control FOL in tomato plants, opening new avenues of research for the valorization of BSFL frass.

**Abstract:**

This study investigated the effect of black soldier fly larvae (BSFL) frass derived from BSFL reared on a diet composed of fruit/vegetable/bakery/brewery residues (FVBB diet) and on the Gainesville diet (GV diet) on the development of tomato (*Solanum lycopersicum*) Fusarium wilt caused by *Fusarium oxysporum* f. sp. *lycopersici* (FOL). Tomato plants were grown in a substrate inoculated with FOL that was amended (10%, v:v) or not (control) with either a commercial compost, pasteurized (70 °C for 1 h) frass from BSFL reared on a FVBB diet, non-pasteurized frass from BSFL reared on a FVBB diet, pasteurized frass from BSFL reared on the GV diet, or non-pasteurized frass from BSFL reared on the GV diet. The results show that frass from BSFL reared on the GV diet, irrespective of pasteurization, inhibited FOL root colonization and reduced the severity of tomato Fusarium wilt to a far greater extent than frass from BSFL reared on a FVBB diet and commercial compost made of peat, seaweed, and shrimps. This study suggests that BSFL frass, depending on the larval rearing diet, has the potential to serve as a pasteurized or non-pasteurized soil amendment with prophylactic properties against FOL in tomato plants, opening new avenues of research for the valorization of BSFL frass.

## 1. Introduction

Black soldier fly (*Hermetia illucens*) larvae (BSFL) frass, the residual material resulting from the bioconversion of organic matter by BSFL, is gaining attention as potential organic amendment for plant growth [1,2]. Larval frass is a rich source of organic material, which makes it a valuable amendment to improve soil fertility [3,4]. Studies have reported the potential of BSFL frass to either partially or completely replace the use of commercial inorganic fertilizers in food crops such as sweet potato (*Ipomoea batatas*) [5], maize (*Zea mays*) [6,7], lettuce (*Lactuca sativa*) [8], basil (*Ocimum basilicum*) [9], Swiss chard (*Beta vulgaris*) [10], kale (*Brassica oleracea* var. *sabellica*) [11,12], komatsuna (*Brassica rapa* var. *perviridis*) [13], winter wheat (*Triticum aestivum*) [14], French beans (*Phaseolus vulgaris*), and tomato (*Solanum lycopersicum*) [11]. Moreover, the use of frass derived from different types of diets has been shown to increase phenol/antioxidant capacity, crude protein and fiber contents, stem diameter, aerial mass, shoot length, yield, and mannose, magnesium, potassium, manganese, sodium, and zinc contents in leaves [7,9,10,12,13,14].

Besides improving soil fertility and plant growth, BSFL frass was recently shown in vitro to have antifungal activity against different fungal plant pathogens, including *Botrytis cinerea*, *Alternaria solani*, *Fusarium oxysporum*, *Sclerotinia sclerotiorum*, and *Rhizoctonia solani* [1,15], attributed to the microorganisms inhabiting it, suggesting that BSFL frass could suppress the development of fungal plant diseases.

The objectives of this study were (1) to investigate the potential of BSFL frass to suppress Fusarium wilt in tomato plants caused by *Fusarium oxysporum* f. sp. *lycopersici* (FOL) and (2) to evaluate the impact of larval rearing diet and pasteurization on the suppressive effect of BSFL frass.

## 2. Materials and Methods

### 2.1. Diets Preparation

BSFL were reared on two different diets. The diet labeled as the FVBB (fruit/vegetable/bakery/brewery) diet had a humidity level of 70% and consisted of a combination of fruits, vegetables, bakery items, and spent brewer’s grains. The FVBB diet was composed, in fresh weight, of 39% fresh fruits (pineapple (5%), cantaloupe (2%), orange (7%), apple (3%), grape (2%), strawberry (2%), bell pepper (7%), tomato (5%), lemon (2%), banana (2%), and pear (2%)), 36% fresh vegetables (lettuce (10%), carrot (3%), cabbage (3%), onion (2%), leek (3%), celery (3%), broccoli (3%), cauliflower (2%), potato (5%), and corn (2%)), 15% bread, and 10% spent brewer’s grains. The fresh fruits, vegetables, and bread, purchased from a local supplier (Tout Prêt Inc., Sainte-Foy, Québec, QC, Canada), were shredded using an industrial food processor (Rietz disintegrator, model: RA2-8-K322; Bepex Company, TX, USA) and blended together with spent brewer’s grains in a tank (Qualtech model: DSC12336, Company Qualtech, Saint-Hyacinthe, QC, Canada) until a homogeneous mixture was achieved. The Gainesville (GV) diet, known as a reference diet for house flies, consisted, in fresh weight, of 50% wheat bran (Rudolph, Québec, QC, Canada), 30% alfalfa meal (La Coop, Lévis, QC, Canada), and 20% cornmeal (Shah Trading Company, Montréal, QC, Canada) mixed at a humidity of 70% prior to feeding [16].

### 2.2. Frass Preparation and Pasteurization

The fly colony was maintained in cages at the Laboratoire de Recherche en Sciences Environnementales et Médicales (LARSEM) of Université Laval (Québec, QC, Canada). Corrugated cardboard strips containing egg clutches obtained from the fly cages were suspended above 160 g of each diet in containers measuring 17.5 cm × 19 cm. These containers were covered with double agricultural netting and left undisturbed for 24 h. Subsequently, the cardboard strips were removed, and the containers were placed in an incubator (27 °C, 70% relative humidity) with a 12 h photoperiod for a duration of 5 days. During the first five days, the larva substrate was kept moist. After hatching, a total of 800 five-day-old larvae were manually counted and transferred into containers, along with 800 g of their respective new diets at 70% humidity. The rearing of BSFL continued for 10 days (required time to reach 40% of prepupal stage) under conditions of 27 °C, 70% relative humidity, and complete darkness. Subsequently, frass (residual after harvesting larvae) was collected by sieving in a sterile environment. Half of the frass sample was poured into a sterile bottle, sealed, and subjected to a pasteurization process by heating it at 70 °C for 1 h in a water bath.

### 2.3. FOL

FOL was provided by the Laboratoire d’Expertise et de Diagnostic en Phytoprotection (Ministère de l’Agriculture, des Pêcheries et de l’Alimentation, Québec, QC, Canada). The fungus was cultivated at 24 °C in a flask containing potato dextrose broth (100 mL; Becton, Dickinson and Company, Sparks, MD, USA) on a rotary shaker (150 rpm) for one week; five potato dextrose agar (PDA; Becton, Dickinson and Company) disks (5 mm) covered with actively growing mycelium were used to inoculate the flask. Mycelium was recovered using cheesecloth and then homogenized with a blender in sterile distilled water containing NaCl (0.5%; physiological water). The fungal suspension was then adjusted to a concentration of 1 × 10^4^ propagules mL^−1^ of physiological water using a hemocytometer.

### 2.4. Tomato Seeds

Tomato seeds (cultivar M82) were surface-sterilized by soaking in a 0.6% sodium hypochlorite solution for 10 min, followed by a 70% ethanol solution for 30 s, and then washed three times with sterile distilled water.

### 2.5. Effect of Frass on FOL Root Colonization and Tomato Fusarium Wilt Severity

The growth medium (PRO-MIX BX; Premier Tech, Rivière-du-Loup, QC, Canada) was autoclaved at 121 °C for 60 min and was then transferred into UV-sterilized pots (300 mL). Pots were amended (10%, v:v) or not (control) with either non-pasteurized GV frass, non-pasteurized FVBB frass, pasteurized GV frass, pasteurized FVBB frass, or commercial compost (Sea Compost, Fafard, Québec, QC, Canada). Surface-sterilized tomato seeds were then sown in each pot (one seed per pot) and grown in a greenhouse located at Université Laval. The greenhouse was maintained at day and night temperatures of 22 °C and 20 °C, respectively, with a relative humidity ranging from 40% to 50%. Supplemental lighting was provided for 16 h each day, with a photosynthetic photon flux density (PPFD) of 180 μmol m^−2^ s^−1^ from 1000 W HPS lamps. The substrate was drenched with 100 mL of FOL suspension per pot (1 × 10^4^ propagules mL^−1^) three times (four, six, and eight weeks after sowing). The experiment was conducted as a randomized block design with three replicate pots per treatment.

Fusarium wilt severity was assessed by calculating the percentage of leaves displaying typical symptoms (wilted, yellow-colored leaves) in relation to the total number of leaves for each plant [17]. One week after the first FOL inoculation (five weeks after sowing), disease severity was rated weekly for a period of 42 days to construct disease progress curves. The trapezoidal integration method was employed to calculate the area under the disease progress curve (AUDPC) [18]. At 49 days post inoculation (dpi), the tomato plants were harvested and the root colonization by FOL was determined following the procedure described by Chérif et al. [19]. Briefly, washed roots (3 g) were blended in sterile distilled water (30 mL). Serial dilutions were prepared and 200 μL of each dilution was plated on PDA supplemented with 200 mg L^−1^ tetracycline (EMD Biosciences Inc., La Jolla, CA, USA). After four days of incubation in the dark at 24 °C, FOL colonies were counted on PDA to determine the number of colony forming units (CFU) per gram of fresh root.

### 2.6. Analyses of Variance

Statistical analyses were conducted as described in Barrada et al. [20]. Analyses of variance (ANOVAs) were performed on the data using R (R-4.1.1, R Core Team, 2021, Vienna, Austria). Data were transformed using Tukey’s ladder of powers to improve the normality of data. Results were presented as the mean of three replicates ± standard error. When significant (*p* < 0.05), treatment means were compared using post hoc LSD test.

## 3. Results

**Figure 1:**
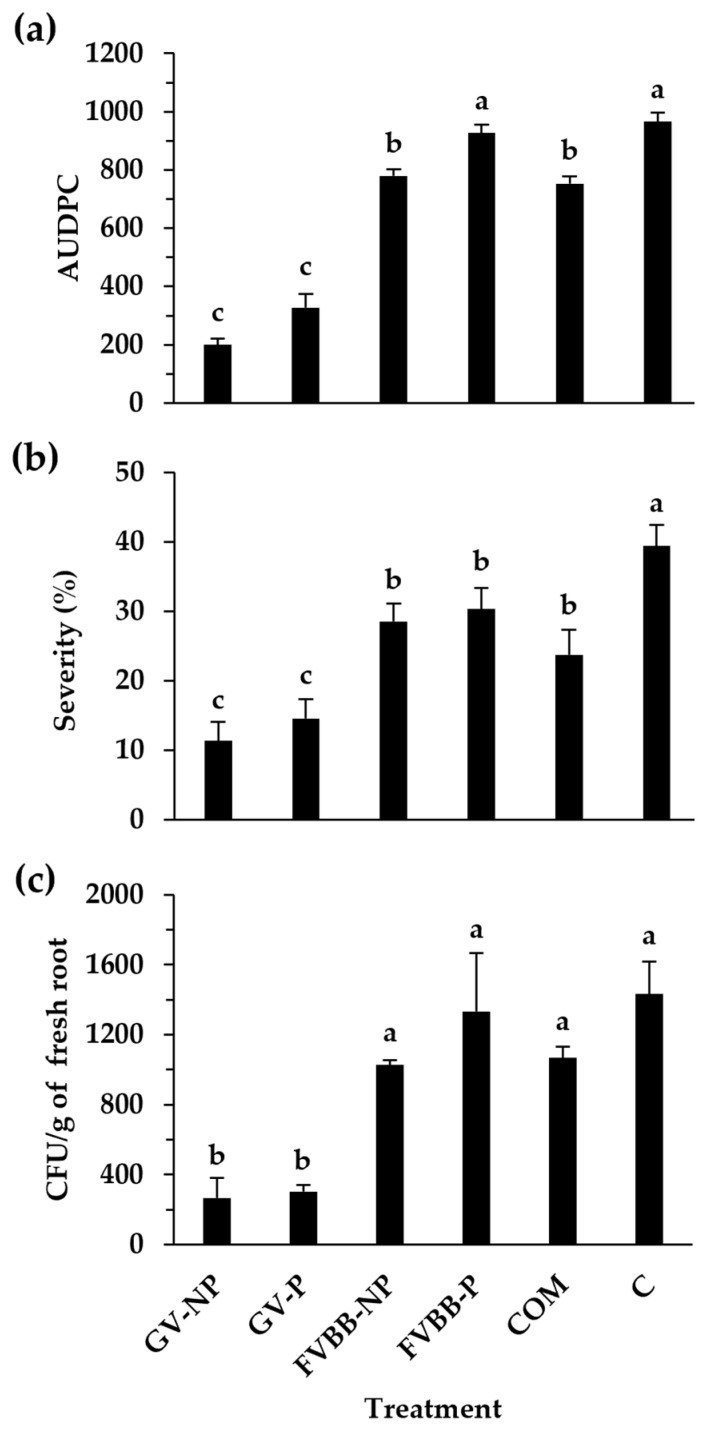
Effect of pasteurized (P) and non-pasteurized (NP) frass derived from two black soldier fly larvae diets (Gainesville (GV) and fruit/vegetable/bakery/brewery (FVBB)) and commercial compost (COM) on tomato Fusarium wilt area under the disease progress curve (AUDPC) (**a**), Fusarium wilt severity rated at 49 days post inoculation (**b**), and on the number of *Fusarium oxysporum* f. sp. *lycopersici* (FOL) colony forming units (CFU) per gram of fresh root (**c**). Control (C) tomato plants were grown in a substrate containing no frass/compost. Each value represents the mean of three replicates ± standard error. The means sharing the same letter are not significantly different according to the post hoc LSD test (*p* < 0.05).

The effect of compost and frass on Fusarium wilt AUDPC and severity rated at 49 dpi is presented in Figure 1a,b, respectively. The incorporation of frass from BSFL reared on the GV diet (pasteurized or non-pasteurized) and compost significantly (*p* < 0.05) reduced Fusarium wilt AUDPC (Figure 1a) and severity rated at 49 dpi (Figure 1b) as compared to the control. The incorporation of frass from BSFL reared on the GV diet (pasteurized or non-pasteurized) resulted in significantly lower Fusarium wilt AUDPC and severity rated at 49 dpi as compared to frass from BSFL reared on a FVBB diet (pasteurized or non-pasteurized). Frass from BSFL reared on the GV diet (pasteurized or non-pasteurized) significantly reduced the root colonization by FOL, expressed as the number of CFU per g of fresh root (Figure 1c), while compost and frass from BSFL reared on a FVBB diet showed no significant effects.

## 4. Discussion

Previous work conducted by our group showed that GV and FVBB frass have N-P-K, macronutrient, and micronutrient contents comparable to those in commercially available organic fertilizers [1] and that GV and FVBB frass were as efficient as commercially available compost in promoting plant growth [21]. Regarding the maturity of the final product used, C/N ratios of GV and FVBB frass were 25.4 and 19.1, respectively [1]. Both values are suitable for a mature compost, as reported by de Bertoldi et al. [22] and Arabzadeh et al. [1].

In the present study, frass derived from BSFL reared on the GV diet strongly reduced FOL root colonization of tomato plant as well as Fusarium wilt severity rated at 49 dpi and expressed as AUDPC, which considers the cumulative disease severity over the entire time period. The suppressive effect of GV diet frass against FOL could result from the direct antifungal activity of microorganisms present in the frass. It has previously been reported that the microorganisms present in GV frass represent one of the key factors contributing to its antifungal activity [1,15]. The suppressive effect of frass against FOL could also result from the stimulation of plants’ natural defenses by microorganisms or chemicals present in frass. BSFL frass was reported to stimulate systemic resistance in plants due to the presence of beneficial microbes or eliciting molecules as chitin [3,23].

Whatever the underlying mechanism of the suppressive effect of frass from the GV diet against FOL, the results obtained herein show that the suppressive effect was not significantly affected by the pasteurization process (70 °C for 1 h). This is of particular importance considering that the newly established EU regulations include insect frass in a new category named “insect excrements”, with the requirement that frass to be used as a fertilizer has to be subjected to heat treatment at 70 °C for 1 h [4].

This study revealed that frass derived from BSFL reared on the GV diet, irrespective of pasteurization, inhibited FOL root colonization and reduced the severity of tomato Fusarium wilt to a far greater extent than frass from BSFL reared on a FVBB diet and compost made of peat, seaweed, and shrimps. These findings suggest that BSFL frass, depending on the larval rearing diet, has the potential to serve as a pasteurized or non-pasteurized soil amendment with prophylactic properties against FOL in tomato plants, opening new avenues of research for the valorization of BSFL frass.

## Data Availability

The data presented in this study are available upon request from the corresponding author.
